# Identifying Heat Waves in Florida: Considerations of Missing Weather Data

**DOI:** 10.1371/journal.pone.0143471

**Published:** 2015-11-30

**Authors:** Emily Leary, Linda J. Young, Chris DuClos, Melissa M. Jordan

**Affiliations:** 1 School of Natural Resources and Environment, University of Florida, PO Box 116455, Gainesville, FL, 32611, United States of America; 2 Department of Statistics, University of Florida, PO Box 118545, Gainesville, FL, 32611, United States of America; 3 Public Health Research Unit, Florida Department of Health, 4052 Bald Cypress Way, Tallahassee, FL, 32399-1708, United States of America; University of Washington, UNITED STATES

## Abstract

**Background:**

Using current climate models, regional-scale changes for Florida over the next 100 years are predicted to include warming over terrestrial areas and very likely increases in the number of high temperature extremes. No uniform definition of a heat wave exists. Most past research on heat waves has focused on evaluating the aftermath of known heat waves, with minimal consideration of missing exposure information.

**Objectives:**

To identify and discuss methods of handling and imputing missing weather data and how those methods can affect identified periods of extreme heat in Florida.

**Methods:**

In addition to ignoring missing data, temporal, spatial, and spatio-temporal models are described and utilized to impute missing historical weather data from 1973 to 2012 from 43 Florida weather monitors. Calculated thresholds are used to define periods of extreme heat across Florida.

**Results:**

Modeling of missing data and imputing missing values can affect the identified periods of extreme heat, through the missing data itself or through the computed thresholds. The differences observed are related to the amount of missingness during June, July, and August, the warmest months of the warm season (April through September).

**Conclusions:**

Missing data considerations are important when defining periods of extreme heat. Spatio-temporal methods are recommended for data imputation. A heat wave definition that incorporates information from all monitors is advised.

## Introduction

Numerous public health studies have used weather data for the 108 cities included in the National Morbidity, Mortality, and Air Pollution Study (NMMAPS) (e.g. [1–3]). These studies have been important to identifying regional differences in health effects based on climate indicators (e.g. [1, 4]). Although some have found no evidence of regional differences in health effects due to heat waves [5], others have found differences so great that a significant adverse effect of climate on health can be observed in one region while, in another, no effect or a protective effect is seen [1, 6]. Multiple studies have concluded that meteorological thresholds for weather (generally temperature) should be region-specific or local (e.g. [7–8]).

One source of confusion is the lack of a uniform definition of a heat wave used in climate-change research studies. Currently, the National Weather Service (NWS) initiates heat alert procedures when the heat index is expected to exceed 40.56°C–43.33°C (105°F-110°F) for at least 2 consecutive days [9]. The Intergovernmental Panel on Climate Change [10] defined a heat wave to be the longest period within a year composed of at least 5 consecutive days with maximum temperatures at least 5°C higher (approximately 9°F higher) than the climatology of the same calendar day. However, this becomes challenging when different sources use differing numbers of years to define the climatology.

For health studies, the percentiles of the year-round daily maximum temperature for the study period have been used to define a heat wave as the longest period of consecutive days that satisfy three conditions: (1) the daily maximum temperature must be above the 97.5 percentile for *at least* 3 days, (2) the daily maximum temperature must be above the 81 percentile for *every day*, and (3) the *average* of daily maximum temperature for the consecutive period must be above the 97.5 percentile, (e.g. [1, 11–12]).

Different geographic regions have different timing and durations for both warm and cold seasons. For instance, Florida is known for its year-round warm weather and has many seasonal residents escaping cold seasons by flocking to Florida. Defining a warm season in Florida is problematic because, relative to other parts of the U.S., the warm season could be considered to include the entire year. The Florida Climate Center (FCC), within the Center for Ocean-Atmospheric Prediction Studies, defines the Florida warm season to be from April through September, which is when the highest temperatures and humidity levels tend to occur and is the definition used here [13].

Some research papers have considered and compared different definitions of heat waves, exploring associations between heat waves and public health measures and have found conclusions can change based on which definition of a heat wave is utilized [2–3, 7]. Further, associations between extreme heat and health risks depend upon the thresholds used to define a heat wave, making it important to choose these *a priori* [3].

When assessing the effect of heat waves on public health, missing weather data issues are rarely discussed [1, 4, 8, 11, 12, 14–17]. Missing data are typically ignored if 8% or less of the total amount of data are missing (e.g. [3, 6, 18, 19]), if at least 18 hourly readings occur during a day [20], or if a geographical area has data for at least half of a month for each month studied [21]. Most studies have either removed weather stations with missing data or completed the study with no added processing of meteorological data (e.g. [6, 18]). Only Deschênes and Greenstone [22] specifically discussed imputation for missing meteorological data when assessing the effect of missing data on the association between exposure and US annual county mortality rates, using data from 1968 to 2002. In that study, only weather monitors with no missing data were included in the analysis. Inverse distance weighting of temperature measurements from monitors within 200 km of each county’s centroid was used to predict county-level temperatures. Analyses were conducted using subsets of the monitor data as well as multiple imputation. The conclusions were the same for all approaches considered.

In this paper, the primary objective is to develop a heat wave definition with direct application to public health research for Florida. Historical weather records are described, modeled, and used to define heat waves within the state of Florida. Methodologies to account for missing weather records are developed and missing data imputed for use in identifying heat waves. The effects of missing data on heat wave definitions are discussed. All calculations were completed using Fahrenheit measurements, but have been converted to Celsius measurements for publication purposes.

## Materials and Methods

### Weather Data

The FCC receives data from the National Climatic Data Center weather monitors and runs multiple data quality checks while computing additional indicators, such as heat index. Heat index is calculated using the Rothfusz equation and adjustments, as is currently used by the National Oceanic and Atmospheric Administration (NOAA) [23]. The FCC data are NOAA data that are further processed for accuracy and quality. The NWS currently initiates heat alerts using heat index, specifically when the heat index is expected to exceed 40.56°C–43.33°C (105°F-110°F). Although other approaches exist, the public health literature tends to use temperature, heat index, apparent temperature, or some combination of these to determine an extreme heat event [1–3, 6, 7, 11, 12, 14–16]. Given these facts and the prevalence of high humidity, especially during Florida’s warm season, heat index is used here as the measure of heat (e.g. [8, 18, 24]).

The warm season heat indexes collected from 1973–2012 for 43 FCC weather monitors are used in this study. The use of 40 years of warm season data should provide more precise estimates of the percentiles than the typical 10–20 years of data used in other public health studies (e.g. [25–27]). The Florida Department of Health (FDOH) is interested in the effect of heat waves on morbidity and mortality. Although data from the billing records for Medicare and Medicaid recipients would have provided morbidity data for an extended period of time, the more complete billing data from all Florida hospitals and emergency departments, with the exception of state-operated, Federal, and Shriner’s hospitals, are used here. However, these health records on heat-related morbidity are only available from 2005 through 2012. Thus, 40 years of heat index values are used for precise estimation of the percentiles of maximum daily heat index, and the estimated percentiles are used to identify heat waves occurring from 2005 through 2012.

### Heat Wave Definition

Considering Florida’s hot and humid climate, the daily maximum heat index was thought to be a better single measure of heat than temperature. With the FDOH’s interest in serving all of Florida, initial interest focused on defining state-wide heat waves. Florida spans over six degrees latitude and seven degrees longitude, and the heat index often varies widely across the state at any point in time. In fact, from 2005 through 2012, there was no three-day period for which all 43 monitors had a heat index above their respective 50^th^ percentiles. Consequently, no state-wide heat wave could be identified and was not considered further.

**Fig 1:**
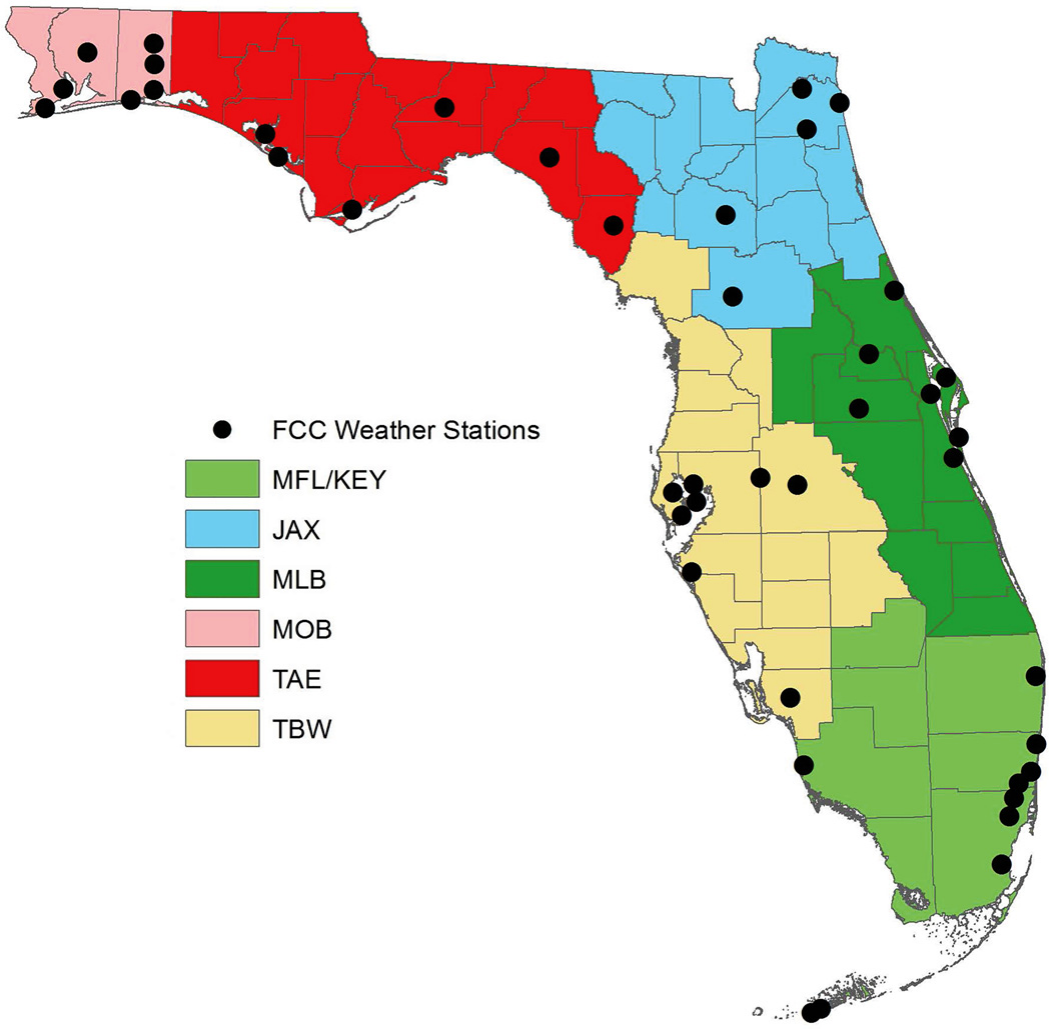
National Weather Service regions and locations of FCC monitors within Florida.

Although meteorological thresholds for weather could be local or region-specific [3, 4, 8], the use of a local or monitor-specific definition of heat wave would exclude many rural and agricultural areas, important target populations for Florida’s public health services. Regional heat waves were considered using the seven National Weather Service (NWS) regions in Florida (Fig 1). In this analysis, the Keys region (KEY) was combined with the Miami region (MFL), resulting in six regions. Using NWS regions provides an inherent method for communicating extreme heat alerts through the NWS alert system, a major interest of FDOH.

According to Meehl and Tebaldi [11], global climate models indicated that heat waves will become more frequent and of a longer duration. Their simulations indicate that the mean number of heat waves for the Chicago area, the only North American city named in their manuscript, will increase from the current average of 1.09 to 2.17 per year, to an average of 1.63 to 2.44 per year, over the next 90 years. Anderson and Bell [6] studied heat waves in 43 U.S. cities from 1987 to 2005. Their results indicated that, on average, each city experienced 1.9 heat waves per year. Based on these simulations and historical results, it is reasonable to investigate heat waves within each Florida NWS region from 2005 to 2012, the time period for which complete morbidity data exists for Florida.

The heat wave definition used here is based upon that in Peng et al. [12] and Bobb et al. [1] and incorporates the practical considerations, such as data accessibility, from Barnett et al. [7]. For a period to be considered a regional heat wave, each monitor in the region must (1) have the daily maximum heat index above the 80^th^ warm season percentile and (2) have at least three days, which need not be consecutive, in the period above a regional upper threshold. Two approaches for defining the regional upper threshold were evaluated. The first used an upper percentile of daily maximum heat index and the second was a regional benchmark. Other studies have examined percentile thresholds in their heatwave definitions and those results informed the percentiles used here [1, 3, 6, 12, 17, 28–30].

A regional benchmark is an absolute threshold representing an actual measured value of the upper heat index for defining extreme heat for an entire region. The regional benchmark value used was the highest daily maximum heat index resulting in at least one heat wave during the period of interest (2005–2012).

### Missing data

Some of the daily maximum heat index values are missing, with more data missing during cooler months within the warm season (April, May, September) than in warmer months within the warm season (June, July, August). Further, some monitors have more missing data than others (S1 Table). These differences primarily result from varying decisions being made as to the frequency with which data are to be recorded for a monitor and are not due to the monitors themselves. Because extreme heat is more likely to occur in the warmer months, missing data in these months are of particular concern.

All weather data for the 43 weather monitors were checked for data errors and summarized for quality and heat index calculated using the Rothfusz equation and adjustments, the method used by the National Weather Service [23]. Warm-season percentiles of daily maximum heat index for each station were calculated and used to define heat waves for each region within Florida, using four approaches. First, as is commonly done, missing data were ignored. To determine regional percentiles when ignoring missing data, the warm season daily heat index values from recorded monitors within a region were averaged, and the regional percentiles of these daily averages determined. In addition to (1) ignoring missing data, three different imputation models were considered: (2) a temporal model, (3) a spatial model, and (4) a spatio-temporal model.

To impute missing data using a temporal model, the daily maximum heat indexes associated with each of the 43 FCC weather monitors were modeled, and each monitor’s model was used to impute missing heat index data for that monitor. The integrated nested Laplace approximation (INLA) method used here provides a computational advantage over the standard MCMC (Markov Chain Monte Carlo) approaches [31, 32]. No study has used this type of model to impute heat index, but Haslett et al. [33] have used a Bayesian methodology to reconstruct prehistoric climates using fossil data.

Specifically, the following Bayesian model of daily maximum heat index was fit using the R-INLA package from R [34].

$$y_{t|i}=\;f_{1}\left(x_{1\;t|i}\right)+\;f_{2}\left(x_{2\;t|i}\right)+\;f_{3}\left(x_{3\;t|i}\right)+e_{t|i}$$ (1)

where *y*_*t|i*_ is the maximum heat index for monitor *i* on day *t; x*_1 *t*|*i*_ is the date associated with day *t* for monitor *i*; *x*_2 *t*|*i*_ is the year associated with day *t* for monitor *i*; *x*_3 *t*|*i*_ is the day of the year for day *t* associated with monitor *i*; and e_*t|i*_ ~ *N*(0,*σ* ^2^) is the random error of the model. *x*_1 *t*|*i*_ is modeled using a first-order autoregressive model (AR(1)), *x*_2 *t*|*i*_ and *x*_3 *t*|*i*_ are modeled using independent random walk models of order 2 (RW2) [31]. Note that date and day of the year are not equivalent; date is used in the model to represent the time from the first day of the warm season in 1973 to the end of the warm season in 2012, whereas day of year is the Julian Day. Within this temporal model, the AR(1) model of date is used to capture long-term temporal changes in exposure, the RW2 model of year is used to capture yearly exposure trends, and the RW2 model with day of year (Julian Day) is used to capture exposure trends within a year.

For 13 monitors’ models, the Hessian was not positive definite, even after tolerance levels were re-adjusted, due to the lack of a year effect. Thus, for these monitors, the effect of year was not included in the model.

The second imputation method used ordinary kriging, an interpolation method used for predicting spatial data. For this model, spatial relationships for daily maximum heat index were modeled to impute missing heat index data. By computing the weighted average of the given observed data values in a defined neighborhood of those values, the value of a function at a certain location can be predicted. We assume second order stationarity and isotropy in these data, and an exponential covariance model was chosen based on a sample of heat index data from different months and years within the warm season. From these data, the exponential covariance model was the best fit, using AIC. Ordinary kriging assumes a spatial mean, which is assumed here to be unknown and constant for any time *t*. Universal kriging was not appropriate as the data displayed no consistent trend at the scale of the modeling, over the period of interest. Thus, the variation in the heat index at time *t* is captured in the correlation structure. Specifically, for each day *t*, *y*_*it*_, the daily maximum heat index for monitor *i*, was modeled as a spatial process:

$$\;y_{i|t}=\;\mu_{t}+e_{i|t}$$ (2)

where *μ*_*t*_ represents the overall mean and *e*_*i|t*_ is the random error, assumed to have an exponential covariance structure, which captures the spatial covariance among monitors on day *t*. The models were fit using restricted maximum likelihood (REML), and model predictions provided imputed values for missing data. Ordinary kriging was not possible if less than two monitors recorded daily maximum heat index across Florida, for a specific day. In these cases, monitor-specific monthly averages, across all years, were used as the imputed values. This occurred for less than 3% of the days in the study period (n = 163; N = 7320), none of which occurred in the warmer months of the warm season, June, July or August, and none were associated with periods of high daily maximum heat indexes.

Time series models incorporate information from the same monitor over time; spatial models consider information from surrounding monitors from the same day. The third imputation approach, spatio-temporal models, is based on both spatial relationships and time trends. Using REML, the space-time process for daily maximum heat index for monitor *i* on day *t*, *y*_*it*_, was fit using the following spatio-temporal model:

$$y_{it}=\beta_{0}+\;\beta_{1}y_{i,t-1}+e_{it}$$ (3)

where *β*_0_ represents the intercept; *β*_1_ is an unknown parameter; *y*_*i*,*t*−1_ represents the lag effect of heat index on day *t* for monitor *i*; and *e*_*it*_ represents the random error, assumed to have an exponential covariance structure among monitors within each day.

**Fig 2:**
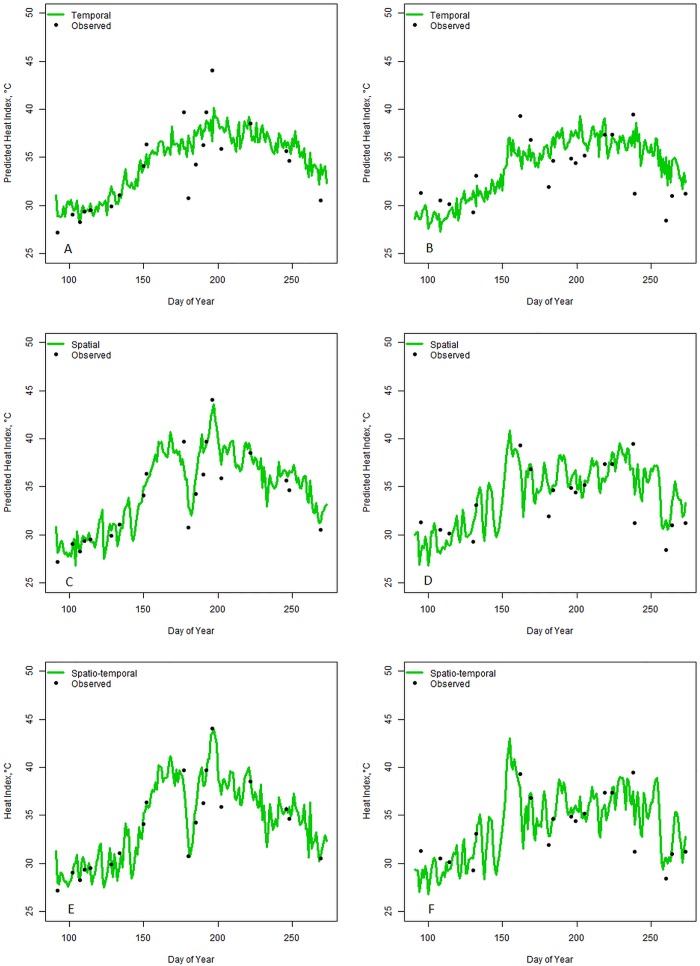
Imputation model results for the Gainesville weather monitor within the Jacksonville (JAX) region, for the years 1981 and 1985, using each imputation method. Imputed daily maximum heat indexes during the warm season are graphed (by day) in green with observed values dropped from the sample denoted by black dots. (A) Graph of the temporal model results for 1981. (B) Graph of the temporal model results for 1985. (C) Graph of the spatial model results for 1981. (D) Graph of the spatial model results for 1985. (E) Graph of the spatio-temporal model results for 1981. (F) Graph of the spatio-temporal model results for 1985.

To assess how well the temporal, spatial, and spatio-temporal models predicted missing daily maximum heat index values, a 10% stratified sample of daily maximum heat index measurements for all stations during the warm season for the 40 year period was taken (314,760 total observations where strata were the day, with 7320 possible days). The models for each method were fit without these sampled data, and the predicted values were compared with the observed values (Fig 2). The primary objective is to identify the method that is best able to predict missing values and, given that the observed values are available from the 10% sample, the root mean squared prediction error (RMSPE) for the sample was calculated for each model. In addition, because extreme heat is of primary interest, RMSPE was also calculated for data that were greater than 37.78°C (100°F). For extreme heat, the 97.5, 95, 90, and 80^th^ percentiles are often considered (e.g. [3, 11–12]). The 97.5, 95, 90, and 80^th^ percentiles for daily maximum heat index values during the warm season were estimated using the complete data (observed and imputed), derived from each of the four missing data approaches and all 40 years of data.

## Results

### Missing data imputation

Unlike in Deschênes and Greenstone [22], all of the 43 FCC weather monitors had some missing data during 1973–2012, making an analysis based only on monitors with complete data impossible. The missing data approach affects the estimates of the upper percentiles and thus the identified heat waves.

When comparing the model predictions for the 10% stratified sample not included during model development to their observed values, the spatio-temporal model had the lowest RMSPE and the lowest RMSPE for those heat index values greater than 37.78°C (100°F) for 41 of the 43 monitors (S2 Table). The spatial model performed the next best. The temporal model produced the worst fit for 33 of the 43 monitors and was less able to predict unusual heat events, compared to the spatial and spatio-temporal (Fig 2). Although the RMSPE tended to be greater for the spatial model compared to the spatio-temporal model, the modeled daily maximum heat index had similar shapes, with better modeling of extremes compared to the temporal model predictions.

### Heat Waves

Heat waves were identified for the six NWS regions using each missing data approach. Specifying a specific percentile as an upper threshold for defining a heat wave proved challenging, at least partly because the range of heat indexes between the 80^th^ and 97.5 percentiles is narrow (Table 1 and S3 Table). In contrast, Lippman et al. [27] used a reference set for the calculated incidence rate ratios of heat-related morbidity, which corresponded to daily mean temperatures between 15.56°C and 21.11°C (60°F and 70°F), and all of their analyses were conducted using increments of 10°F (approximately 5.56°C). Similarly, in Fletcher et al. [25], odds ratios between temperature and hospital admissions for acute renal failure were quantified for approximately 5°F (approximately 2.78°C) changes of minimum, mean, and maximum temperature and heat index. Here, the differences in the 80^th^ to 97.5 percentiles are less than 5.5°C (10°F) for all monitors (S3 Table). As can be seen in Table 1, for monitor 722046 in the MLB region, the 80th percentile is 34.53°C and the 97.5th percentile is 35.31°C, a difference of approximately 0.78°C. Consequently, defining heat waves using percentiles as the upper threshold was problematic. Thus, a regional benchmark was considered.

**Table 1:** JAX and MLB 97.5 and 80^th^ percentiles (°F) for max daily heat index for regionally aggregated, ignoring missing data, and three imputation methods.

Region	Monitor/ region	Ignore Missing		Temporal		Spatial		Spatio-temporal		% miss JJA^a^
		80	97.5	80	97.5	80	97.5	80	97.5	
JAX	regional	37.89	41.89	—	—	—	—	—	—	0.12
	722055	36.83	39.83	36.27	39.28	37.00	39.74	37.06	40.17	0.47
	722060	38.17	41.94	38.00	41.83	38.00	41.83	38.00	41.83	0.01
	722065	38.11	42.50	37.89	42.08	37.89	42.06	37.89	42.08	0.01
	722066	37.89	42.50	37.00	41.94	37.17	41.89	37.17	41.94	0.08
	722146	37.61	41.00	37.39	41.00	37.39	41.00	37.39	41.00	0.01
MLB	regional	37.94	41.94	—	—	—	—	—	—	0.27
	722040	38.78	43.50	38.17	42.81	38.23	42.72	38.17	42.80	0.15
	722046	35.50	39.28	34.53	35.31	37.46	39.96	37.33	39.97	0.92
	722050	37.61	40.33	37.61	40.33	37.61	40.33	37.61	40.33	0.00
	722056	37.39	40.44	37.17	40.39	37.17	40.39	37.17	40.39	0.01
	722057	38.28	42.56	37.89	42.11	38.00	42.11	37.94	42.11	0.13
	747946	39.11	42.33	37.61	40.97	37.75	41.78	37.94	41.97	0.52
	747950	37.44	40.94	37.01	40.67	37.17	40.67	37.17	40.67	0.14

^a^Note: “% miss JJA” denotes the percent missing data during the warm months of the warm season, June, July and August.

In Tong et al. [8], for 1996–2005 in Brisbane, Australia, the 95^th^ percentile for maximum temperature in the summer was 34.1°C (93.38°F) and was 32.7°C (90.86°F) for the entire year. In Fletcher et al. [25], the maximum temperatures used to determine associations between hospitalizations for acute renal failures and temperatures in the state of New York were less than 28.89°C (84°F, ascertained using a table). In contrast, for this study, the 80^th^ percentile of every monitor’s daily maximum heat index was at least 34.44°C (94°F) and some were as high as 39.28°C (102.7°F). A full comparison of percentiles determined here to those found in other studies is challenging because many publications do not provide estimates of the percentiles used to define a heat wave. Sometimes, but not always, the percentiles can be inferred from graphics or tables (e.g. [2–3, 6]).

Regional benchmark values used as the upper threshold had a smaller range of values, from 37.78°C (100°F) to 44.44°C (112°F), compared to the 97.5 percentiles for daily maximum heat index, which varied from 35.33°C (95.6°F) to 43.94°C (111.1°F). The regional benchmark, the highest daily maximum heat index resulting in at least one heat wave during 2005–2012, was used to identify extreme heat within Florida’s warm season. All NWS regions were analyzed and heat waves identified within each region. However, the Jacksonville (JAX) region and Melbourne (MLB) region were chosen to illustrate results and facilitate discussion (for all NWS region results, see S4 Table).

#### JAX region

Regional benchmark values of 41.67°C (107°F), ignoring missing data, and of 40°C (104°F), imputing missing data, were used to identify one heat wave for NWS region JAX, which includes the city of Jacksonville in Florida’s northeast. The heat wave was defined to be from August 5 to August 14, 2007, when missing data were ignored and from August 6 to August 11, 2007, when using each of the imputation methods.

#### MLB region

The MLB NWS region covers the east-central area of Florida. For the MLB region, the regional benchmark differed with the method used to handle missing data. When ignoring missing data, the regional benchmark was 41.11°C (106°F), whereas it was 37.78°C (100°F) using the temporal model and 38.89°C (102°F) when using the spatial or spatio-temporal model to impute missing data. When using the temporal model to impute data, three heat waves were identified, all of them occurring in 2010: June 14 to June 16; July 24 to August 1; and August 17 to August 21. Two completely different heat waves were identified using the spatial and spatio-temporal methods of handling missing data: August 13, 2005 to August 20, 2005 and June 20, 2009 to June 22, 2009. When ignoring missing data, two heat waves were identified, one of which was similar to that identified using the temporal model (but lasting two additional days): July 24 to August 3, 2010 and August 11 to August 17, 2011.

## Discussion

Days are more likely to have high maximum heat index values during the warmer months of the warm season. Thus, it is not surprising that the percent of missing data during the warmer months tend to accentuate differences in the upper percentiles obtained from imputation. Although the heat wave definition that ignores missing data is easy to calculate and no imputation is performed, the heat waves defined do not always represent extreme heat for all monitors, and thus all areas, within the region. Typically, a few monitors within a region are able to influence the daily averages, which in turn affects the corresponding percentiles. Monitor percentiles obtained when ignoring missing data tend to be larger than the corresponding percentiles using imputed data, regardless of imputation method used. Requiring all monitors to exceed a regional benchmark ensures that the entire region is experiencing a heat wave, which may not be the case when the average is used. For these reasons, missing data should not be ignored.

When using the temporal model, the predictions of daily maximum heat index, and consequently the percentiles, tend toward the mean as the amount of missing data increases. Thus, the time series imputation method is not appropriate when the goal is accurate estimation of upper percentiles. Models that used spatial information provide better predictions of daily maximum heat index during periods of extreme heat. As a result, the spatial models provide more precise predictions of daily maximum heat index and, consequently, better estimates of the percentiles of daily maximum heat index. Thus, spatial information should be incorporated in predictions of missing heat index.

The spatio-temporal model includes both the information from surrounding monitors as well as the temporal trends for a particular monitor. Through its predictions, the model can reflect the fact that a monitor tends to record higher (or lower) heat index values than its neighbors. Thus, it is not surprising that the spatio-temporal model provided better predictions than the spatial model that does not include the temporal information from a particular monitor. Although no adjustments were made for technological advances in weather monitoring equipment or any weather monitor movement, these perceived issues should have little or no impact on the specific heat waves considered in recent years but can affect any calculated thresholds using historical data that might be used to define recent heat waves. Issues such as urban sprawl, with the well-established heat-island effect, and the effect of including agricultural areas may also influence heat definitions. Although close to larger bodies of water, coastal areas in Florida generally have a coastal breeze that may alleviate or balance out the additional humidity, compared to inland climates [35]. Thus, no adjustments of this type were made in this analysis.

The estimated percentiles are the foundation for identifying heat waves, and missing daily maximum heat index affects the percentiles used in heat wave definitions. Basing the percentiles on the warm season and not the full year causes the upper percentiles to be larger and the differences in the 80^th^ and 97.5 percentiles to be smaller.

The method used to impute missing daily maximum heat index values can influence the heat wave period identified through the imputed values themselves or through the effect on the percentiles used to define extreme heat. To identify a heat wave using imputed data, all monitors had to have daily maximum heat index values above the 80^th^ percentile, ensuring the entire region was experiencing extreme heat. In contrast, when ignoring missing data and identifying heat waves using a regional average, only the average had to exceed the regional benchmark. Thus, August 5 and 12 in the JAX region (S4 Table), were included in the heat wave when missing data were ignored, but not otherwise. Similarly, the heat wave defined from June 20–22, 2009, for the MLB region using the spatial and spatio-temporal imputed data could not be identified as a heat wave using either the temporal model or when ignoring missing data. Because the imputed daily maximum heat index tended toward the mean for the temporal imputation model, the imputed values were consistently less than their respective 80^th^ percentile, making it less likely to identify a heat wave when data were missing.

Missing data increases the uncertainty associated with identifying heat waves. As noted earlier, ignoring missing data can lead to substantial errors in identifying heat waves. Imputation can inform identification of heat waves, but can also result in errors. If one monitor in a region records a high daily maximum heat index and the remaining monitors in the region fail to record heat index values for that day, information from other monitors in the state is used to inform imputation for the missing data. This could lead to correct conclusions about the presence of a heat wave, or it could either incorrectly identify a heat wave (imputed heat index was above that present at the monitor) or fail to identify a heat wave (imputed heat index was below that present at the monitor). Given that imputation tends to miss the extremes, it is more likely that heat waves are not identified using the imputed values.

Better predictions with few drawbacks lead to the recommendation that the spatio-temporal method be used to model and predict missing daily maximum heat index values. This model is appealing because temporal and spatial components are incorporated, both important considerations for weather-related research analyses. If this method were not feasible, the spatial model would be the next best choice for imputing missing data. The temporal model and ignoring missing data are inferior to the models incorporating a spatial component and should not be used.

## Conclusions

This study highlights challenges in creating a general methodology to identify periods of extreme heat for Florida. The NWS regions were utilized because the weather is relatively uniform within each region. The regional approach to a methodology allowed meaningful heat wave definitions and also an inherent method to communicate heat alerts to the public. The heat wave definition considered here can be used for each NWS region in Florida and can also be applied to other areas outside of Florida. Although many studies have looked at relationships between heat waves and health, most do not consider missing weather data. For future studies, it is recommended to use a spatio-temporal model to impute missing values, leading to more precise estimates of percentiles and more accurate identification of heat waves.

## Supporting Information

S1 Table Proportion of missing daily maximum heat index values for each weather monitor. (DOCX)

S2 Table RMSPE for all values and for those daily maximum heat index values over 37.78°C 100°F), overall and by weather monitor for each method of data imputation: temporally, spatially, and spatiotemporal. Lower values indicate a better fit of the model to the data.
(DOCX)

S3 Table 97.5, 95, 90, and 80^th^ percentiles for the warm season from 1973–2012, by monitor and method of imputation, in degrees Celsius. (DOCX)

S4 Table Heat waves and regional thresholds identified for all NWS regions from 2005–2012, by region and method of imputation. (DOCX)

## References

[1] Bobb JF, Dominici F, Peng RD. A Bayesian Model Averaging Approach for Estimating the Relative Risk of Mortality Associated with Heat Waves in 105 U.S. Cities. Biometrics. 2011 12; 67: 1605–1616. 10.1111/j.1541-0420.2011.01583.x 21447046 PMC3128186

[2] Gasparrini A, Armstrong B. The Impact of Heat Waves on Mortality. Epidemiology. 2011 1; 22(1): 68–73. 10.1097/EDE.0b013e3181fdcd99 21150355 PMC3324776

[3] Barnett AG, Hajat S, Gasparrini A, Rocklöv J. Cold and Heat Waves in the United States. Environ Res. 2012 1; 112: 218–224. 10.1016/j.envres.2011.12.010 22226140

[4] Curriero F, Heiner HS, Samet J, Zeger S, Strug L, Patz J. Temperature and Mortality in 11 Cities of the Eastern United States. Am J Epidemiol. 2002; 155(1): 80–87. 11772788 10.1093/aje/155.1.80

[5] Davis RE, Knappenberger PC, Michaels PJ, Novicoff WM. Seasonality of climate–human mortality relationships in US cities and impacts of climate change. Clim Res. 2004; 26: 61–76.

[6] Anderson GB, Bell ML. Heat Waves in the United States: Mortality Risk during Heat Waves and Effect Modification by Heat Wave Characteristics in 43 U.S. Communities. Environ Health Perspect. 2011 2;119(2): 210–218. 10.1289/ehp.1002313 21084239 PMC3040608

[7] Barnett AG, Tong S, Clements ACA. What Measure of Temperature is the Best Predictor of Mortality? Environ Res. 2010 6; 110: 604–611. 10.1016/j.envres.2010.05.006 20519131

[8] Tong S, Wang XY, Barnett AG. Assessment of Heat-related Health Impacts in Brisbane, Australia: Comparison of Different Heatwave Definitions. PLoS One. 2010 8; 5(8): e12155, 1–5. 10.1371/journal.pone.0012155 20730050 PMC2921381

[9] National Oceanic and Atmospheric Administration, National Weather Service [Internet]. NOAA's Watch, Warning, and Advisory Products for Extreme Heat. [cited 6 April 2014]. Available from: http://www.nws.noaa.gov/os/heat/index.shtml

[10] Intergovernmental Panel on Climate Change (IPCC) [Internet]. Climate Change 2007: Synthesis Report. Contribution of Working Groups I, II and III to the Fourth Assessment Report of the Intergovernmental Panel on Climate Change [Core Writing Team, Pachauri, R.K and Reisinger, A. (eds.)]. [accessed 6 April 2014]. IPCC, Geneva, Switzerland, 104pp. Available from http://www.ipcc.ch/publications_and_data/ar4/syr/en/spms1.html

[11] Meehl GA, Tebaldi C. More Intense, More Frequent, and Longer Lasting Heat Waves in the 21st Century. Science. 2004 8; 305: 994–997. 15310900 10.1126/science.1098704

[12] Peng RD, Bobb JF, Tebaldi C, McDaniel L, Bell ML, Dominici F. Toward a Quantitative Estimate of Future Heat Wave Mortality under Global Climate Change. Environ Health Perspect. 2011 5; 119(5): 701–706. 10.1289/ehp.1002430 21193384 PMC3094424

[13] Winsberg MD, Simmons M [Internet]. An Analysis of the Beginning, End, Length, and Strength of Florida’s Warm Season. Florida Climate Center. [accessed 6 April 2014]. Available from: http://climatecenter.fsu.edu/topics/specials/floridas-hot-season

[14] Kent ST, McClure LA, Zaitchik BF, Smith TT, Gohlke JM. Heat Waves and Health Outcomes in Alabama (USA): The Importance of Heat Wave Definition. Environ Health Perspect. 2014; 122 (2) 151–158. 10.1289/ehp.1307262 24273236 PMC3914868

[15] Pentavou K, Theoharatos G, Mavrakis A, Santamouris M. Evaluating thermal comfort conditions and health responses during an extremely hot summer in Athens. Build Environ. 2011 46: 339–344.

[16] Huth R, Kyselý J, Pokorná L. A GCM Simulation of Heat Waves, Dry Spells, and their Relationships to Circulation. ‎Clim. Change. 2000 46: 29–60.

[17] Medina-Ramón M, Schwartz J. Temperature, temperature extremes, and mortality: a study of acclimatization and effect modification in 50 US cities. Occup Environ Med. 2007; 64(12): 827–833. 17600037 10.1136/oem.2007.033175 PMC2095353

[18] Rocklöv J, Ebi K, Forsberg B. Mortality related to temperature and persistent extreme temperatures: A study of cause-specific and age-stratified mortality. Occup Environ Med. 2011; 68: 531–536. 10.1136/oem.2010.058818 20962034

[19] Rocklöv J, Forsberg B, Meister K. Winter Mortality Modifies the Heat-Mortality Association the Following Summer. Eur Respir J. 2009; 33: 245–251. 10.1183/09031936.00037808 18799511

[20] Barreca AI. Climate Change, Humidity, and Mortality in the United States. J Environ Econ Manage 2012; 63: 19–34. 25328254 10.1016/j.jeem.2011.07.004 PMC4199665

[21] Basu R, Feng WY, Ostro B. Characterizing Temperature and Mortality in Nine California Counties. Epidemiology. 2008 1; 19(1): 138–145. 18091422 10.1097/EDE.0b013e31815c1da7

[22] Deschênes O, Greenstone M. Climate Change, Mortality, and Adaptation: Evidence from Annual Fluctuations in Weather in the US. Am Econ J Appl Econ 2011 10; 3: 152–185.

[23] National Weather Service [Internet]. The Heat Index Equation. [accessed 3 June 2015] Available from: http://www.wpc.ncep.noaa.gov/html/heatindex_equation.shtml

[24] Sung TI, Wu PC, Lung SC, Lin CY, Chen MJ, Su HJ. Relationship Between Heat Index and Mortality of 6 Major Cities in Taiwan. Sci Total Environ. 2013; 442: 275–281. 10.1016/j.scitotenv.2012.09.068 23178831

[25] Fletcher BA, Lin S, Fitzgerald EF, Hwang S. Association of Summer Temperatures With Hosptial Admissions for Renal Diseases in New York State: A Case-Crossover Study. Am J Epidemiol. 2012 175(9):907–916. 10.1093/aje/kwr417 22455834

[26] Anderson GB, Dominici F, Wang Y, McCormack MC, Bell ML, Peng RD. Heat-related Emergency Hospitalizations for Respiratory Diseases in the Medicare Population. Am J Respir Crit Care Med. 2013 5; 187: 1098–1103. 10.1164/rccm.201211-1969OC 23491405 PMC3734617

[27] Lippman S, Fuhrmann C, Waller A, Richardson D. Ambient Temperature and Emergency Department Visits for Heat-related Illness in North Carolina, 2007–2008. Environ Res. 2013; 124: 35–42. 10.1016/j.envres.2013.03.009 23643292

[28] Hajat S, Armstrong B, Baccini M, Biggeri A, Bisanti, Russo A et al Impact of High Temperatures on Mortality: Is There an Added Heat Wave Effect? Epidemiology. 2006 11; 17 (6): 632–638. 17003686 10.1097/01.ede.0000239688.70829.63

[29] Anderson BG, Bell ML. Weather-Related Mortality How Heat, Cold, and Heat Waves Affect Mortality in the United States. Epidemiology 2009 3; 20 (2): 205–213. 10.1097/EDE.0b013e318190ee08 19194300 PMC3366558

[30] Nitschke M, Tucker GR, Hansen AL, Williams S, Zhang Y, Peng B. Impact of two recent extreme heat episodes on morbidity and mortality in Adelaide, South Australia: a case-series analysis. Environ Health 2011 10(42). 10.1186/1476-069X-10-42 PMC3116460 21592410

[31] Rue H, Martino S, Chopin N. Approximate Bayesian Inference for Latent Gaussian Models Using Integrated Nested Laplace Approximations. J R Statist Soc B. 2009; 71(2): 319–392.

[32] Rue H, Martino S. Approximate Bayesian inference for hierarchical Gaussian Markov random field models. J. Statist. Plann. Inference. 2007 137:3177–3192.

[33] Haslett J, Whiley M, Bhattacharya S, Salter-Townshend M, Wilson SP, Allen JRM, Huntley B, Mitchell FJG. Bayesian palaeoclimate reconstruction. J. R. Stat. Soc. Ser. A. 2006 169(3): 395–438.

[34] Blangiardo M, Cameletti M, Baio G, Rue H. Spatial and Spatio-Temporal models with R-INLA. Spat Spatio-temporal Epidemiol. 2013 3; 4: 33–49 10.1016/j.sste.2012.12.001 23481252

[35] Zierden D, Griffin M [press release] Humidity in Florida. [accessed 25 May 2014] Available from: http://climatecenter.fsu.edu/topics/humidity

