# Supplementary material for: Identifying Heat Waves in Florida: Considerations of Missing Weather Data
Source: PLoS One. 2015 Nov 30;10(11):e0143471. doi: 10.1371/journal.pone.0143471 (PMC4664249; doi:10.1371/journal.pone.0143471)
Supplement: S1 Table — (DOCX) [file pone.0143471.s001.docx]

S1 Table. Proportion of missing daily maximum heat index values for each weather monitor.

| Monitor | Monitor Name | APR,MAY,SEP | JUN,JUL,AUG |
| --- | --- | --- | --- |
| 722055 | OCALA INTL J TAYLOR | 0.55 | 0.47 |
| 722060 | JACKSONVILLE/INTNL. | 0.25 | 0.01 |
| 722065 | JACKSONVILLE NAS | 0.22 | 0.01 |
| 722066 | MAYPORT NS | 0.38 | 0.08 |
| 722146 | GAINESVILLE RGNL | 0.18 | 0.01 |
| 722010 | KEY WEST/INT. | 0.09 | 0.00 |
| 722015 | KEY WEST NAS | 0.09 | 0.00 |
| 722020 | MIAMI | 0.09 | 0.00 |
| 722024 | OPA LOCKA | 0.67 | 0.62 |
| 722025 | FORT LAUDERDALE HOL | 0.11 | 0.02 |
| 722026 | HOMESTEAD ARB | 0.24 | 0.14 |
| 722030 | WEST PALM BEACH/IN | 0.20 | 0.08 |
| 722037 | NORTH PERRY | 0.70 | 0.68 |
| 722038 | NAPLES MUNI | 0.46 | 0.40 |
| 722049 | POMPANO BEACH AIRPAR | 0.68 | 0.63 |
| 722040 | MELBOURNE REGIONAL | 0.29 | 0.15 |
| 722046 | SPACE COAST RGNL | 0.96 | 0.92 |
| 722050 | ORLANDO INTL AP | 0.12 | 0.00 |
| 722056 | DAYTONA BEACH INTL | 0.24 | 0.01 |
| 722057 | ORLANDO SANFORD | 0.28 | 0.13 |
| 747946 | NASA SHUTTLE LANDING | 0.66 | 0.52 |
| 747950 | PATRICK AFB/COCOA B | 0.36 | 0.14 |
| 722210 | VALPARAISO/EGLIN AF | 0.56 | 0.28 |
| 722215 | BOB SIKES | 0.25 | 0.03 |
| 722221 | PENSACOLA REGIONAL AP | 0.55 | 0.31 |
| 722225 | PENSACOLA NAS | 0.38 | 0.01 |
| 722226 | WHITING FLD NAS NORT | 0.30 | 0.04 |
| 722246 | EGLIN AF AUX NR 3 D | 0.45 | 0.22 |
| 747770 | HURLBURT FIELD (AF) | 0.50 | 0.14 |
| 722120 | CROSS CITY/CROSS CI | 0.35 | 0.25 |
| 722140 | TALLAHASSEE MUNICIP | 0.20 | 0.01 |
| 722200 | APALACHICOLA MUNI | 0.56 | 0.27 |
| 722224 | PERRY FOLEY | 0.78 | 0.71 |
| 722245 | PANAMA CITY BAY CO | 0.52 | 0.34 |
| 747750 | TYNDALL AFB | 0.47 | 0.14 |
| 722104 | ALBERT WHITTED | 0.59 | 0.52 |
| 722106 | FORT MYERS/PAGE FLD | 0.15 | 0.09 |
| 722110 | TAMPA INTL AIRPORT | 0.13 | 0.00 |
| 722115 | SARASOTA BRADENTON | 0.17 | 0.03 |
| 722116 | ST PETERSBURG CLEAR | 0.13 | 0.01 |
| 722119 | LAKELAND LINDER RGN | 0.38 | 0.30 |
| 722123 | BARTOW MUNI | 0.51 | 0.46 |
| 747880 | MACDILL AFB/TAMPA | 0.24 | 0.13 |
