# Supplementary material for: Identifying Heat Waves in Florida: Considerations of Missing Weather Data
Source: PLoS One. 2015 Nov 30;10(11):e0143471. doi: 10.1371/journal.pone.0143471 (PMC4664249; doi:10.1371/journal.pone.0143471)
Supplement: S2 Table — Lower values indicate a better fit of the model to the data. (DOCX) [file pone.0143471.s002.docx]

**S2 Table.** **RMSPE for all values and for those daily maximum heat index values over 37.78°C 100°F), overall and by weather monitor for each method of data imputation: temporally, spatially, and spatiotemporal.** Lower values indicate a better fit of the model to the data.

|  | Temporal | | Spatial | | Spatio-temporal | |
| --- | --- | --- | --- | --- | --- | --- |
| Monitor | RMSPE | RMSPE > 37.78°C | RMSPE | RMSPE > 37.78°C | RMSPE | RMSPE > 37.78°C |
| 722055 | 3.28 | 1.52 | 3.12 | 0.86 | 2.30 | 0.72 |
| 722060 | 4.74 | 2.73 | 3.49 | 1.72 | 2.07 | 1.29 |
| 722065 | 4.91 | 2.97 | 3.76 | 2.07 | 2.42 | 1.61 |
| 722066 | 5.02 | 3.10 | 4.33 | 2.07 | 2.89 | 1.55 |
| 722146 | 3.83 | 2.08 | 3.33 | 1.45 | 2.08 | 1.08 |
| 722010 | 3.54 | 1.87 | 3.73 | 2.69 | 2.04 | 1.46 |
| 722015 | 3.79 | 2.31 | 4.18 | 2.99 | 2.42 | 1.68 |
| 722020 | 3.63 | 1.83 | 3.09 | 1.69 | 1.83 | 0.90 |
| 722024 | 3.56 | 1.90 | 2.38 | 1.63 | 1.68 | 1.02 |
| 722025 | 4.12 | 2.38 | 3.76 | 2.53 | 2.62 | 1.77 |
| 722026 | 4.35 | 2.64 | 3.70 | 2.58 | 2.41 | 1.66 |
| 722030 | 3.69 | 1.89 | 2.78 | 1.54 | 1.71 | 0.94 |
| 722037 | 3.43 | 1.78 | 2.11 | 1.33 | 1.52 | 0.83 |
| 722038 | 3.85 | 2.11 | 3.46 | 1.99 | 2.54 | 1.23 |
| 722049 | 3.37 | 1.75 | 2.01 | 1.10 | 1.45 | 0.65 |
| 722040 | 3.89 | 2.39 | 3.95 | 3.13 | 3.00 | 2.50 |
| 722046 | 3.46 | 1.82 | 2.28 | 0.99 | 2.13 | 0.98 |
| 722050 | 3.96 | 2.08 | 2.79 | 1.32 | 1.95 | 0.99 |
| 722056 | 4.21 | 2.20 | 3.13 | 1.04 | 2.09 | 0.79 |
| 722057 | 4.55 | 2.63 | 3.96 | 2.59 | 3.03 | 2.10 |
| 747946 | 4.00 | 2.33 | 2.76 | 1.77 | 2.26 | 1.56 |
| 747950 | 4.04 | 2.32 | 3.50 | 1.62 | 2.74 | 1.24 |
| 722210 | 4.45 | 2.61 | 3.62 | 1.64 | 2.40 | 1.32 |
| 722215 | 4.78 | 2.70 | 3.97 | 1.68 | 2.28 | 1.06 |
| 722221 | 4.55 | 2.69 | 3.57 | 1.92 | 2.23 | 1.22 |
| 722225 | 4.30 | 2.61 | 4.47 | 2.84 | 2.72 | 1.97 |
| 722226 | 5.16 | 3.07 | 4.38 | 2.31 | 2.70 | 1.60 |
| 722246 | 4.88 | 2.97 | 4.10 | 2.11 | 2.90 | 1.69 |
| 747770 | 4.98 | 3.09 | 4.15 | 2.27 | 2.67 | 1.65 |
| 722120 | 3.69 | 2.06 | 3.20 | 1.71 | 2.16 | 1.27 |
| 722140 | 3.92 | 2.13 | 3.49 | 1.52 | 2.03 | 0.98 |
| 722200 | 4.59 | 2.67 | 3.28 | 1.40 | 2.17 | 1.03 |
| 722224 | 3.65 | 1.99 | 2.27 | 1.07 | 1.69 | 0.92 |
| 722245 | 4.50 | 2.76 | 3.52 | 2.09 | 2.59 | 1.67 |
| 747750 | 4.89 | 3.02 | 3.92 | 2.34 | 2.59 | 1.74 |
| 722104 | 3.27 | 1.92 | 2.82 | 2.02 | 2.27 | 1.53 |
| 722106 | 3.90 | 2.16 | 3.69 | 2.52 | 2.41 | 1.66 |
| 722110 | 4.00 | 2.16 | 3.04 | 1.78 | 1.94 | 1.20 |
| 722115 | 4.40 | 2.69 | 4.36 | 3.43 | 3.09 | 2.48 |
| 722116 | 4.05 | 2.58 | 4.73 | 3.87 | 3.53 | 3.00 |
| 722119 | 3.96 | 2.17 | 2.92 | 1.60 | 2.20 | 1.25 |
| 722123 | 3.77 | 2.10 | 2.74 | 1.46 | 2.05 | 1.16 |
| 747880 | 4.22 | 2.47 | 3.70 | 2.49 | 2.86 | 1.90 |
| Overall | 4.15 | 2.39 | 3.49 | 2.08 | 2.38 | 1.50 |
