# Supplementary material for: Identifying Heat Waves in Florida: Considerations of Missing Weather Data
Source: PLoS One. 2015 Nov 30;10(11):e0143471. doi: 10.1371/journal.pone.0143471 (PMC4664249; doi:10.1371/journal.pone.0143471)
Supplement: S3 Table — (DOCX) [file pone.0143471.s003.docx]

**S3 Table.** **97.5, 95, 90, and 80^th^ percentiles for the warm season from 1973-2012, by monitor and method of imputation, in degrees Celsius**.

|  | | Ignoring Missing Data | | | | Temporal | | | | Spatial | | | | Spatio-temporal | | | |
| --- | --- | --- | --- | --- | --- | --- | --- | --- | --- | --- | --- | --- | --- | --- | --- | --- | --- |
| Region | Monitor | 80 | 90 | 95 | 97.5 | 80 | 90 | 95 | 97.5 | 80 | 90 | 95 | 97.5 | 80 | 90 | 95 | 97.5 |
| JAX | 722055 | 36.83 | 37.94 | 39.28 | 39.83 | 36.27 | 36.93 | 37.94 | 39.28 | 37.00 | 38.11 | 39.09 | 39.74 | 37.06 | 38.16 | 39.30 | 40.17 |
|  | 722060 | 38.17 | 39.61 | 41.00 | 41.94 | 38.00 | 39.56 | 40.44 | 41.83 | 38.00 | 39.56 | 40.44 | 41.83 | 38.00 | 39.56 | 40.44 | 41.83 |
|  | 722065 | 38.11 | 39.56 | 41.00 | 42.50 | 37.89 | 39.28 | 40.67 | 42.08 | 37.89 | 39.28 | 40.67 | 42.06 | 37.89 | 39.28 | 40.67 | 42.08 |
|  | 722066 | 37.89 | 39.50 | 41.00 | 42.50 | 37.00 | 38.81 | 40.33 | 41.94 | 37.17 | 38.83 | 40.33 | 41.89 | 37.17 | 38.94 | 40.39 | 41.94 |
|  | 722146 | 37.61 | 38.83 | 40.22 | 41.00 | 37.39 | 38.72 | 39.67 | 41.00 | 37.39 | 38.72 | 39.67 | 41.00 | 37.39 | 38.72 | 39.67 | 41.00 |
| MFLK | 722010 | 38.72 | 39.56 | 40.39 | 40.67 | 38.61 | 39.50 | 40.39 | 40.67 | 38.56 | 39.50 | 40.39 | 40.67 | 38.61 | 39.50 | 40.39 | 40.67 |
|  | 722015 | 38.11 | 39.56 | 40.39 | 41.33 | 38.11 | 39.50 | 40.39 | 41.22 | 38.11 | 39.50 | 40.39 | 41.22 | 38.11 | 39.50 | 40.39 | 41.22 |
|  | 722020 | 37.89 | 38.72 | 39.56 | 40.33 | 37.61 | 38.72 | 39.56 | 40.33 | 37.61 | 38.72 | 39.56 | 40.33 | 37.61 | 38.72 | 39.56 | 40.33 |
|  | 722024 | 38.11 | 39.28 | 40.61 | 42.00 | 37.67 | 38.12 | 39.11 | 39.61 | 37.89 | 38.83 | 39.56 | 40.61 | 37.94 | 38.72 | 39.39 | 40.61 |
|  | 722025 | 38.11 | 39.50 | 40.33 | 41.06 | 38.00 | 39.28 | 40.33 | 41.00 | 38.00 | 39.28 | 40.33 | 41.00 | 38.00 | 39.28 | 40.33 | 41.00 |
|  | 722026 | 38.67 | 39.61 | 40.67 | 42.11 | 38.00 | 39.39 | 40.64 | 42.00 | 38.11 | 39.50 | 40.67 | 42.00 | 38.17 | 39.50 | 40.67 | 42.00 |
|  | 722030 | 38.00 | 38.78 | 39.61 | 40.39 | 37.61 | 38.72 | 39.56 | 40.33 | 37.61 | 38.72 | 39.56 | 40.33 | 37.61 | 38.72 | 39.56 | 40.33 |
|  | 722037 | 38.11 | 39.22 | 39.61 | 40.61 | 37.53 | 38.09 | 38.52 | 39.28 | 37.72 | 38.70 | 39.43 | 40.28 | 37.86 | 38.59 | 39.37 | 40.13 |
|  | 722038 | 38.11 | 39.50 | 40.39 | 41.33 | 37.17 | 38.50 | 39.56 | 40.39 | 37.81 | 38.85 | 39.78 | 40.67 | 37.94 | 38.94 | 39.78 | 40.64 |
|  | 722049 | 37.94 | 39.11 | 39.50 | 40.67 | 35.50 | 37.01 | 38.25 | 39.22 | 37.53 | 38.64 | 39.28 | 40.14 | 37.61 | 38.48 | 39.22 | 39.80 |
| MLB | 722040 | 38.78 | 40.39 | 42.00 | 43.50 | 38.17 | 40.11 | 41.33 | 42.81 | 38.23 | 40.22 | 41.33 | 42.72 | 38.17 | 40.22 | 41.33 | 42.80 |
|  | 722046 | 35.50 | 36.83 | 37.94 | 39.28 | 34.53 | 35.13 | 35.27 | 35.31 | 37.45 | 38.47 | 39.30 | 39.96 | 37.33 | 38.44 | 39.29 | 39.97 |
|  | 722050 | 37.61 | 38.72 | 39.61 | 40.33 | 37.61 | 38.67 | 39.56 | 40.33 | 37.61 | 38.67 | 39.56 | 40.33 | 37.61 | 38.67 | 39.56 | 40.33 |
|  | 722056 | 37.39 | 38.72 | 39.61 | 40.44 | 37.17 | 38.28 | 39.56 | 40.39 | 37.17 | 38.28 | 39.56 | 40.39 | 37.17 | 38.28 | 39.56 | 40.39 |
|  | 722057 | 38.28 | 40.17 | 41.22 | 42.56 | 37.89 | 39.56 | 41.00 | 42.11 | 38.00 | 39.58 | 41.00 | 42.11 | 37.94 | 39.56 | 41.00 | 42.11 |
|  | 747946 | 39.11 | 40.61 | 42.17 | 42.33 | 37.61 | 38.50 | 39.53 | 40.97 | 37.75 | 39.11 | 40.57 | 41.78 | 37.94 | 39.22 | 40.61 | 41.97 |
|  | 747950 | 37.44 | 38.83 | 40.28 | 40.94 | 37.00 | 38.42 | 39.50 | 40.67 | 37.17 | 38.72 | 39.61 | 40.67 | 37.17 | 38.61 | 39.50 | 40.67 |
| MOB | 722210 | 37.94 | 39.50 | 40.61 | 41.94 | 37.39 | 38.39 | 39.56 | 41.00 | 37.39 | 38.78 | 40.22 | 41.06 | 37.35 | 38.78 | 40.33 | 41.50 |
|  | 722215 | 37.72 | 39.28 | 40.67 | 41.94 | 37.39 | 39.00 | 40.33 | 41.89 | 37.39 | 39.00 | 40.33 | 41.83 | 37.39 | 39.00 | 40.33 | 41.89 |
|  | 722221 | 38.17 | 40.22 | 41.22 | 42.56 | 37.39 | 38.72 | 40.33 | 41.83 | 37.61 | 39.06 | 40.39 | 41.89 | 37.61 | 39.24 | 40.58 | 41.94 |
|  | 722225 | 38.72 | 40.39 | 42.06 | 43.56 | 38.11 | 40.22 | 41.83 | 43.28 | 38.11 | 40.22 | 41.83 | 43.28 | 38.11 | 40.22 | 41.83 | 43.28 |
|  | 722226 | 38.11 | 39.67 | 41.22 | 42.83 | 37.61 | 39.53 | 41.06 | 42.56 | 37.72 | 39.56 | 41.06 | 42.56 | 37.61 | 39.56 | 41.06 | 42.56 |
|  | 722246 | 38.11 | 39.61 | 41.83 | 42.61 | 37.17 | 39.00 | 40.61 | 42.00 | 37.61 | 39.28 | 40.61 | 42.00 | 37.39 | 39.28 | 40.67 | 42.00 |
|  | 747770 | 38.17 | 40.22 | 41.89 | 43.17 | 37.39 | 39.22 | 40.67 | 42.22 | 37.61 | 39.28 | 40.67 | 42.17 | 37.44 | 39.28 | 40.91 | 42.33 |
| TAE | 722120 | 38.00 | 39.39 | 40.44 | 41.89 | 37.94 | 39.05 | 40.22 | 41.22 | 37.61 | 38.83 | 40.22 | 41.22 | 37.61 | 38.83 | 40.28 | 41.22 |
|  | 722140 | 37.94 | 39.28 | 40.33 | 41.72 | 37.61 | 39.06 | 40.28 | 41.22 | 37.61 | 39.06 | 40.28 | 41.22 | 37.61 | 39.06 | 40.28 | 41.22 |
|  | 722200 | 38.00 | 39.56 | 40.44 | 41.94 | 37.21 | 38.49 | 39.61 | 41.06 | 37.39 | 38.78 | 40.27 | 41.17 | 37.39 | 38.80 | 40.33 | 41.22 |
|  | 722224 | 37.94 | 39.28 | 40.33 | 41.22 | 35.23 | 36.28 | 37.94 | 39.28 | 37.55 | 38.67 | 39.56 | 40.39 | 37.48 | 38.75 | 39.73 | 40.78 |
|  | 722245 | 38.11 | 39.56 | 41.06 | 42.56 | 37.39 | 38.72 | 40.22 | 41.22 | 37.71 | 39.12 | 40.33 | 41.44 | 37.80 | 39.28 | 40.61 | 42.00 |
|  | 747750 | 38.61 | 40.33 | 41.94 | 43.28 | 37.81 | 39.50 | 40.86 | 42.36 | 37.89 | 39.50 | 40.89 | 42.33 | 37.80 | 39.50 | 41.03 | 42.67 |
| TBW | 722104 | 38.14 | 39.50 | 40.61 | 41.89 | 37.77 | 38.12 | 39.39 | 40.39 | 37.64 | 38.83 | 39.78 | 40.67 | 38.00 | 39.17 | 40.22 | 40.99 |
|  | 722106 | 38.39 | 39.61 | 40.67 | 41.89 | 38.17 | 39.56 | 40.39 | 41.33 | 38.17 | 39.56 | 40.39 | 41.28 | 38.22 | 39.56 | 40.39 | 41.33 |
|  | 722110 | 38.17 | 39.50 | 40.33 | 41.06 | 38.11 | 39.11 | 40.33 | 41.00 | 38.11 | 39.11 | 40.33 | 41.00 | 38.11 | 39.11 | 40.33 | 41.00 |
|  | 722115 | 38.83 | 40.39 | 42.00 | 43.28 | 38.72 | 40.39 | 41.89 | 42.72 | 38.72 | 40.39 | 41.83 | 42.72 | 38.72 | 40.39 | 41.83 | 42.72 |
|  | 722116 | 39.28 | 41.00 | 42.50 | 43.94 | 39.22 | 40.67 | 42.33 | 43.78 | 39.22 | 40.67 | 42.33 | 43.78 | 39.22 | 40.67 | 42.33 | 43.78 |
|  | 722119 | 37.39 | 38.83 | 39.61 | 40.67 | 36.83 | 38.11 | 39.39 | 40.28 | 37.36 | 38.57 | 39.56 | 40.44 | 37.50 | 38.72 | 39.56 | 40.61 |
|  | 722123 | 37.06 | 38.11 | 39.39 | 40.61 | 37.99 | 38.77 | 39.38 | 39.71 | 37.10 | 38.17 | 39.29 | 40.14 | 37.33 | 38.57 | 39.41 | 40.40 |
|  | 747880 | 38.28 | 39.67 | 41.06 | 42.39 | 38.00 | 39.50 | 40.67 | 42.00 | 38.11 | 39.56 | 40.67 | 42.00 | 38.06 | 39.50 | 40.67 | 42.00 |
