# Supplementary material for: Identifying Heat Waves in Florida: Considerations of Missing Weather Data
Source: PLoS One. 2015 Nov 30;10(11):e0143471. doi: 10.1371/journal.pone.0143471 (PMC4664249; doi:10.1371/journal.pone.0143471)
Supplement: S4 Table — (DOCX) [file pone.0143471.s004.docx]

| **S4 Table. Heat waves and regional thresholds identified for all NWS regions from 2005-2012, by region and method of imputation.** | | | |
| --- | --- | --- | --- |
| Region | Imputation Method | Heat Waves | Regional Threshold |
| JAX | Ignoring missing data | August 5-14, 2007 | 41.67°C |
|  | Temporal | August 6-11, 2007 | 40.00°C |
|  | Spatial | August 6-11, 2007 | 40.00°C |
|  | Spatio-temporal | August 6-11, 2007 | 40.00°C |
| MFL/KEY | Ignoring missing data | August 11-21, 2010 | 40.56°C |
|  | Temporal | August 18-21, 2010  July 22-25, 2011 | 39.44°C |
|  | Spatial | August 18-21, 2010  July 22-25, 2011 | 39.44°C |
|  | Spatio-temporal | August 18-21, 2010  July 22-25, 2011 | 39.44°C |
| MLB | Ignoring missing data | July 24-August 3, 2010  August 11-17, 2011 | 41.11°C |
|  | Temporal | June 14-16, 2010  July 24-August 1, 2010  August 17-21, 2010 | 37.78°C |
|  | Spatial | August 13-20, 2005  June 20-22, 2009 | 38.89°C |
|  | Spatio-temporal | August 13-20, 2005  June 20-22, 2009 | 38.89°C |
| MOB | Ignoring missing data | July 20-August 14, 2010 | 44.44°C |
|  | Temporal | July 20-August 14, 2010 | 43.33°C |
|  | Spatial | July 20-August 14, 2010 | 43.33°C |
|  | Spatio-temporal | July 20-August 14, 2010 | 43.33°C |
| TAE | Ignoring missing data | August 5-18, 2007  June 14-23, 2009  July 9-August 10, 2010 | 42.78°C |
|  | Temporal | June 16-23, 2009 | 41.67°C |
|  | Spatial | June 16-23, 2009 | 41.67°C |
|  | Spatio-temporal | June 16-23, 2009  July 28-August 10, 2010 | 41.67°C |
| TBW | Ignoring missing data | June 20-23, 2009 | 41.11°C |
|  | Temporal | August 17-19, 2005  August 1-3, 2010 | 38.33°C |
|  | Spatial | August 16-19, 2005 | 38.89°C |
|  | Spatio-temporal | August 16-19, 2005 | 38.89°C |
